# Caffeine and Bicarbonate for Speed. A Meta-Analysis of Legal Supplements Potential for Improving Intense Endurance Exercise Performance

**DOI:** 10.3389/fphys.2017.00240

**Published:** 2017-05-09

**Authors:** Peter M. Christensen, Yusuke Shirai, Christian Ritz, Nikolai B. Nordsborg

**Affiliations:** ^1^Section of Integrated Physiology, Department of Nutrition, Exercise and Sports, University of CopenhagenCopenhagen, Denmark; ^2^Team DanmarkCopenhagen, Denmark

**Keywords:** performance, supplements, ergogenic aids, intense exercise, running, cycling, rowing, swimming

## Abstract

A 1% change in average speed is enough to affect medal rankings in intense Olympic endurance events lasting ~45 s to 8 min which for example includes 100 m swimming and 400 m running (~1 min), 1,500 m running and 4000 m track cycling (~4 min) and 2,000 m rowing (~6-8 min). To maximize the likelihood of winning, athletes utilizes legal supplements with or without scientifically documented beneficial effects on performance. Therefore, a continued systematic evidence based evaluation of the possible ergogenic effects is of high importance. A meta-analysis was conducted with a strict focus on closed-end performance tests in humans in the time domain from 45 s to 8 min. These test include time-trials or total work done in a given time. This selection criterion results in a high relevance for athletic performance. Only peer-reviewed placebo controlled studies were included. The often applied and potentially ergogenic supplements beta-alanine, bicarbonate, caffeine and nitrate were selected for analysis. Following a systematic search in Pubmed and SportsDiscuss combined with evaluation of cross references a total of 7 (beta-alanine), 25 (bicarbonate), 9 (caffeine), and 5 (nitrate) studies was included in the meta-analysis. For each study, performance was converted to an average speed (km/h) from which an effect size (ES; Cohens d with 95% confidence intervals) was calculated. A small effect and significant performance improvement relative to placebo was observed for caffeine (ES: 0.41 [0.15–0.68], *P* = 0.002) and bicarbonate (ES: 0.40 [0.27–0.54], *P* < 0.001). Trivial and non-significant effects on performance was observed for nitrate (ES: 0.19 [−0.03–0.40], *P* = 0.09) and beta-alanine (ES: 0.17 [−0.12–0.46], *P* = 0.24). Thus, caffeine's and bicarbonate's ergogenic effect is clearly documented for intense endurance performance. Importantly, for all supplements an individualized approach may improve the ergogenic effect on performance.

## Introduction

A 1% change in average speed is enough to affect medal rankings in intense Olympic endurance events lasting ~45 s to 8 min (Table 1). To maximize the likelihood of winning, athletes utilizes legal supplements with or without scientifically documented effects on performance (Tsitsimpikou et al., 2009; Tscholl et al., 2010; Solheim et al., 2016). Extensive narrative reviews exist, but systematic critical evaluation of frequently used supplements effects on human exercise performance are lacking, but are required in order to advise elite-, subelite- and age-group athletes for or against usage. Importantly, the magnitude of possible performance enhancing effects related to supplementation must be seen relative to the obtainable effects of physical, technical and tactical training. When advising for or against supplement usage it is important to realize that early exposure to supplements may increase the likelihood of reverting to illegal performance enhancing strategies (Dodge and Jaccard, 2006). Moreover, the risk of contamination or undeclared illegal substances in products is noteworthy and can result in a positive doping test (Geyer et al., 2004; Outram and Stewart, 2015). Thus, it is important for athletes and staff to balance the potential performance gain with the known risks associated with supplementation.

**Table 1:** **Event duration and average speed for gold winners at the London 2012 and Rio 2016 Olympics in selected intense endurance events for both male and female athletes and the difference in average speed between silver and gold medalists as well as between number 4 and the bronze medalist**.

			**Event duration gold winner (min:s.h)**		**Average speed gold winner (km/h)**		**Difference in speed silver to gold (%)**		**Difference in speed number 4 to bronze (%)**	
		**Distance (m)**	**London 2012**	**Rio 2016**	**London 2012**	**Rio 2016**	**London 2012**	**Rio 2016**	**London 2012**	**Rio 2016**
Running	♂	400	43.94	43.03	32.77	33.47	−1.17	−1.67	−0.60	−0.36
Swimming (Free style)	♂	100	47.52	47.58	7.58	7.57	−0.02	−0.46	−0.08	−0.06
Running	♀	400	49.55	49.44	29.06	29.13	−0.30	−0.14	−0.06	−0.97
Swimming (Free style)	♀	100	53.00	52.70	6.79	6.83	−0.71	0.00	−0.06	−0.09
Running	♂	800	1:40.91	1:42.15	28.54	28.19	−0.81	−0.45	−0.28	−0.46
Swimming (Free style)	♂	200	1:43.14	1:44.65	6.98	6.88	−1.71	−0.52	−0.10	−0.25
Kayak (K1)	♀	500	1:51.456	1:52.494	16.15	16.00	−1.09	−1.60	−0.24	−0.09
Swimming (Free style)	♀	200	1:53.61	1:53.73	6.34	6.33	−1.70	−0.31	−0.01	−0.23
Running	♀	800	1:56.19	1:55.28	24.79	24.98	−0.89	−1.04	−0.05	−0.11
Track cycling (Team pursuit)	♀	3,000/4,000^*^	3:14.051	4:10.236	55.66	57.55	−2.84	−0.88	−0.09	−1.48
Kayak (K1)	♂	1,000	3:26.462	3:31.447	17.44	17.03	−0.34	−0.33	−0.82	−0.18
Running.	♂	1,500	3:34.08	3:50.00	25.22	23.48	−0.33	−0.05	−0.02	−0.02
Track cycling (Team pursuit)	♂	4,000	3:51.659	3:50.265	62.16	62.54	−1.25	−0.32	−0.98	−1.25
Running	♀	1,500	4:10.23	4:08.92	21.58	21.69	−0.07	−0.54	−0.06	−0.21
Rowing (Single sculls)	♂	2,000	6:57.82	6:41.34	17.23	17.94	−0.37	0.00	−0.18	−0.81
Rowing (Single sculls)	♀	2,000	7:54.37	7:21.54	15.18	16.31	−0.70	−0.31	−0.77	−0.08
Mean [95% CI]							−0.89 [−1.25 to 0.53]	−0.54 [−0.79 to 0.29]	−0.28 [−0.44 to 0.12]	−0.42 [−0.64 to 0.19]

*Results were obtained from the official Olympic organization homepage; www.rio2016.com and www.olympic.org (August 2016)*.

* *In female team pursuit distance was 3,000 m in London and 4,000 m in Rio Olympics*.

In the context of detecting subtle differences in performance the type of exercise testing is of outmost importance. To obtain the highest elite-sport relevance (i.e., high ecologic validity), “closed-end” performance tests with a fixed distance (e.g., 2,000 m rowing) or duration (e.g., 6 min maximal rowing) should be the first choice as these resembles competition. However, “open-end” time to exhaustion trials is often applied. This type of evaluation does not resemble athletic competition and likely imposes a high mental stress for the athletes, as there is no set goal. Importantly, closed-end performance tests demonstrate a smaller day to day variance as compared to open-end evaluations (Jeukendrup et al., 1996; Laursen et al., 2007).

The majority of individual Olympic competitions are completed in a time span from ~45 s to 8 min, including 400 m running and 100 m swimming (~1 min) as well as 4,000 m cycling (~4 min) and 2,000 m rowing (~7 min) (Table 1). Thus, the effect on performance from supplementation in this time domain is of high interest. In previous narrative reviews, beta-alanine and sodium-bicarbonate (termed bicarbonate in the present review) (Stellingwerff et al., 2007) as well as caffeine (Davis and Green, 2009) have been proposed to have an ergogenic potential for such athletic events, and recently nitrate supplementation has received growing attention as a potential ergogenic supplement for athletes (Jones, 2014). Consequently, the present systematic meta-analysis evaluates if supplementation with beta-alanine, bicarbonate, caffeine or nitrate can improve intense endurance exercise performance in the time domain 45 s to 8 min when only including closed-end performance tests of high ecological validity.

Currently, a general performance enhancing effect of beta-alanine supplementation on intense exercise is suggested based on mixed exercise protocols encompassing primarily time to exhaustion testing (Artioli et al., 2010; Caruso et al., 2012; Hobson et al., 2012; Quesnele et al., 2014; Trexler et al., 2015). However, a meta-analysis from 2012 including 15 studies with mixed exercise protocols demonstrated no significant effect of supplementation on performance in general, but a separate analysis of 9 mixed performance measures lasting between 60 and 240 s showed a significant effect (Hobson et al., 2012). A later analysis demonstrated that the effect on open-end exercise testing lasting 0.5–10 min was almost five times as high as on performance relevant closed-end testing (Saunders et al., 2017). Thus, there is a need to systematically evaluate the effect of beta-alanine supplementation on exercise performance in trained subjects completing performance relevant testing lasting from 45 s to 8 min.

A number of narrative reviews have addressed the potential ergogenic effect of bicarbonate ingestion (e.g., Requena et al., 2005; McNaughton et al., 2008; Burke, 2013) but meta-analytical approaches have been limited to the effect on running based on only four studies (Schubert and Astorino, 2013) or analyses of different exercise modalities, such as repeated sprinting and time to exhaustion tests (Carr et al., 2011a; Peart et al., 2012). Specifically, a ~2% increase in 1 min mean power sprinting can be expected based on inference statistics but the effect was not reported to be significant at the *P* < 0.05 level (Carr et al., 2011a). Thus, despite conclusions of possible beneficial effects of bicarbonate supplementation for performance the evidence is not convincing and analyses focusing on distinct types of performance tests and time domains are warranted.

Several comprehensive reviews dealing with the potential ergogenic effects of caffeine exist and it is generally acknowledged that ingestion of caffeine can enhance endurance performance lasting 20–250 min (e.g., Graham, 2001; Doherty and Smith, 2004; Ganio et al., 2009; Spriet, 2014,) as well as brief more intense exercise performance lasting 1–3 min (Davis and Green, 2009). However, apart from one review (Ganio et al., 2009) this conclusion is based on a mix of exercise protocols including fixed distance, fixed time or time to exhaustion tests. To the best of our knowledge, only one study has applied a systematic review and meta-analytical approach (Doherty and Smith, 2004) and included various types of exercise tests, resulting in the conclusion that caffeine is less ergogenic during intense short term exercise than longer endurance exercise. Thus, there is a need for a systematic review of caffeines possible ergogenic effects on a single bout of intense exercise lasting 45 s to 8 min.

A recent area of considerable interest from researchers and athletes is nitrate supplementation via beetroot juice or sodium nitrate ingestion. Based on narrative reviews it has been suggested that nitrate supplementation enhances performance (Clements et al., 2014; Jones, 2014) and a meta-analytical approach revealed that a significant effect exists for time to exhaustion tests whereas the effect on time-trials was insignificant (Hoon et al., 2013). Additionally, a current meta-analysis including a high variety of exercise modalities resulted in an unclear effect (Braakhuis and Hopkins, 2015). Thus, systematic analyses of specific time domains and competition relevant testing are lacking.

The primary aim of the present systematic review is to add to previous primarily narrative reviews by including recent studies with beta-alanine, bicarbonate, caffeine, and nitrate supplementation having a strict focus on closed-end performance evaluation in the time domain 45 s to 8 min.

In turn, a secondary aim is to conduct a meta-analysis in which the isolated effects on performance from beta-alanine, bicarbonate, caffeine and nitrate supplementation is evaluated with the same methods allowing for a better comparison of the supplements ergogenic properties, since previous reviews mainly have been centered around a single supplement with various methods applied (e.g., mix of test protocols and large span in time domain).

## Methods

### Criteria for study selection

Two researchers independently identified eligible peer-reviewed studies by a systematic search in the electronic database Medline (PubMed). The search strategy included the following medical subject heading (MeSH) terms: “humans,” “beta-alanine,” “caffeine,” “nitrate,” and “bicarbonate.” MeSH terms were combined with the following wild card strings “run^*^,” “ski^*^,” “swim^*^,” “row^*^,” “kayak^*^” as well as “cycling” and “intense exercise.” A similar search strategy was applied in the database SPORTDiscus. Additional relevant studies were identified from cross-referencing. The search was terminated in April 2016.

Included studies utilized a single or double blind placebo controlled cross-over design, except for studies of beta-alanine where a single blinded parallel group design was allowed due to the long washout period of 6–15 weeks for beta-alanine (Baguet et al., 2009) and in this case data pre and post supplementation were included in the meta-analysis. Only studies applying performance test lasting from 45 s to 8 min with either a fixed duration or fixed distance in able bodied healthy human subjects were included. If several performance tests were conducted in a single study, only the results of the first performance test was included in case at least 20 min of recovery was applied between tests in order to avoid effects related to pacing and fatigue.

Studies were included if they adhered to the present consensus of optimal supplementation. For beta-alanine, the supplementation period should be 28 days or longer with ingestion of ≥3 g per day (Derave et al., 2010). Bicarbonate doses of ≥0.2 g/kg bodyweight ingested >60 min before performance testing as well as multi-day bicarbonate intake of ≥0.3 g/kg daily (McNaughton, 1992a; Siegler et al., 2010, 2012; Carr et al., 2011a) or pre-exercise infusion (Kindermann et al., 1977) was accepted. Caffeine supplementation of ≥2 mg/kg bodyweight supplied 30 min or longer before the performance test (Graham and Spriet, 1995; Pasman et al., 1995; Spriet, 2014) was included. Finally, acute nitrate ingestion of ≥4 mmol, 120–300 min before the performance test (Vanhatalo et al., 2010b; Wylie et al., 2013) or chronic exposure was accepted.

For inclusion in the meta-analysis, performance results were required to be reported as an absolute group mean with a standard deviation or standard error of the mean, both before and after the intervention. In studies in which the standard error were reported, they were converted to standard deviations by multiplying the standard error by the square root of the sample size. Moreover, studies were required to report speed or an entity that was convertible to speed by standard methods (e.g., conversion to speed from time to cover a given distance in field tests or ergometer tests). In one study with rowing as exercise modality only mean power was reported for the 2,000-m distance covered (Carr et al., 2012). This was converted to an average speed from the formula provided by the manufacture of the Concept II rowing ergometer used in that study:

$$\begin{array}{l} \text{Time pr}.\;500\text{m}=3\surd /\left(2.8/\text{Power }\left(\text{watt}\right)\right) \end{array}$$

In studies reporting mean power or total work from ergometer cycling (McNaughton, 1992a,b; McNaughton et al., 1997; Marx et al., 2002; Doherty et al., 2004; Vanhatalo et al., 2010a; Driller et al., 2012; Howe et al., 2013; Hoon et al., 2014a; Thomas et al., 2016) an average speed (v; m/s) was determined numerically using the formula (Martin et al., 1998):

$$\begin{array}{l} \text{Power}\left(\text{watt}\right)=\\ \;\frac{{\left(\left(0,{5\text{CdA}\times \text{Rho}}^{*}{\text{v}}^{2}\right)\;+\;\left({\text{total weight}}^{*}{\text{Crr}}^{*}\text{g}\right)\right)}^{*}\text{v}}{\left(1-\text{drivetrain loss}\%/100\right)} \end{array}$$

In the above calculation it is assumed that no windspeed or slope (gradient) is affecting the rider with the use of fixed values for CdA (drag coefficient × frontal area) = 0.321; Rho (air density) = 1.226 kg/m^3^, total weight (rider and bicycle; kg) using a fixed bike weight of 8 kg and the average body weight listed in each study, Crr (Coefficient of rolling resistance) = 0.005, g (gravitational force constant) = 9.8067 m/s^2^, drivetrain loss% = 3%.

Accordingly, for all studies average speed and the corresponding standard deviation were calculated for each supplement and the placebo (bicarbonate, caffeine, nitrate) or pre supplementation (beta-alanine) condition.

**Figure 1:**
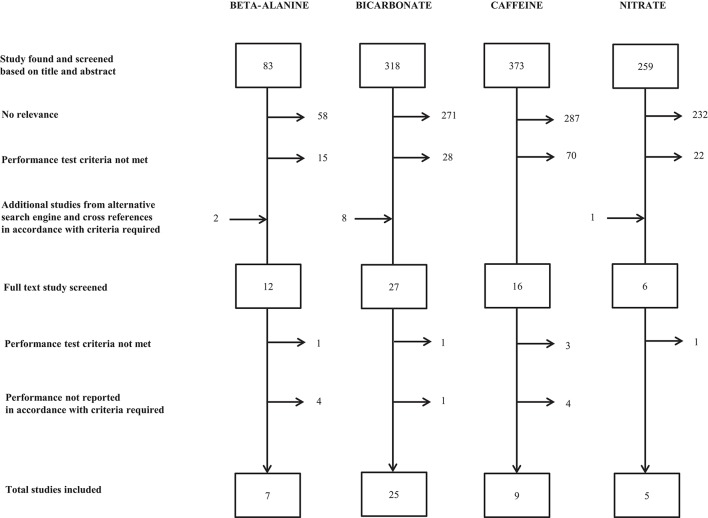
**Flow chart displaying number of included and excluded studies for the evaluation of effects on intense endurance performance lasting 45 s to 8 min following supplementation with beta-alanine, bicarbonate, caffeine and nitrate using Medline (Pubmed) as search engine (see Methods for details regarding study criteria required for inclusion)**.

Based on these criteria a total of 7, 25, 9, and 5 studies of beta-alanine, bicarbonate, caffeine and nitrate, respectively, were included (Figure 1). Some of the included studies applied several performance tests.

### Study quality

**Figure 2:**
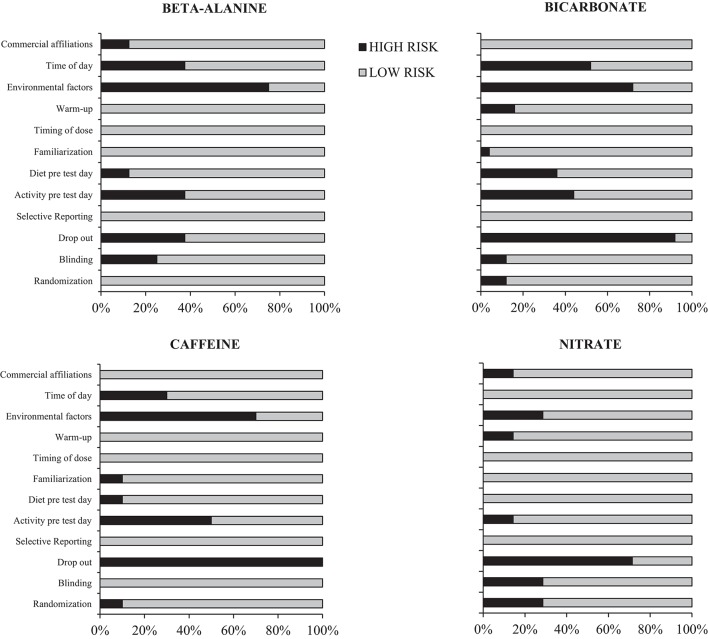
**Quality assessment of studies used for meta-analysis studying the effects on intense endurance performance following supplementation with beta-alanine, bicarbonate, caffeine and nitrate**. Bars denote the relative distribution of studies for each supplements with a high or low risk of bias. See Methods for details regarding criteria for high and low risk.

Based on present guidelines from the Cochrane Institute (Higgins et al., 2011), study quality was assessed with the terms “high risk” or “low risk” of bias modified for exercise performance analyses (Table 2). The relative distribution of studies within each of the four supplements having high or low risk of bias are shown in Figure 2.

**Table 2:** **Definitions for high risk and low risk of bias in studies included in meta-analysis regarding the ergogenic potential from supplementation with beta-alanine, bicarbonate, caffeine and nitrate for increased performance in intense endurance sports**.

	**High risk**	**Low risk**
Commercial affiliations	Mention of affiliations	No commercial affiliations
Time of day	No info provided	Subjects performing at same time of day
Environmental factors	No info provided	Temperature and humidity reported (and wind if outside)
Warm-up	No info provided or free warm-up	Fixed based on a relative intensity or maintained intra-subject warm-up but differences between subjects
Timing of dose	No info provided	Info provided
Test familiarization	No info provided	Mention of familiarization
Diet pre-test day	No info provided	Controlled diet including subjects instructed to eat similar meal
Activity pre-test day	No info provided	Controlled training
Drop out	No info provided	Number of drop outs reported
Blinding	Single Blinded	Double blinded
Randomization	No mention of randomization	Mention of randomization

### Data analysis

Effect sizes (Cohens d) including a Hedges bias correction factor are displayed in forest plots based on standardized differences (SMD) calculated as

(1) $$\begin{array}{r} \text{SMD }=\frac{\left({\text{X}}_{\text{s}}-{\text{X}}_{\text{c}}\right)}{{\text{S}}_{\text{p}}} \end{array}$$

where X_s_ was average speed with supplementation, X_c_ was average speed in the control situation (i.e., Placebo trials for bicarbonate, caffeine and nitrate and pre beta-alanine supplementation), and S_p_ is the pooled standard deviation determined as

(2) $$\begin{array}{r} \text{Sp}=\frac{\text{Squareroot}((\text{SD}{\text{s}}^{2}\;\times \;(\text{n}-1)\pm \text{SD}{\text{c}}^{2}\times (\text{n}-1))}{\left(\text{n}+\text{n}-2\right)} \end{array}$$

where SD_s_ is standard deviation with supplementation, SD_c_ is standard deviation in the control situation and n is the number of subjects.

A multivariate meta-analysis was used to appropriate handle multiple and partly non-overlapping treatments between studies (van Houwelingen et al., 2002). Specifically, a mixed effects model was fitted to logarithm-transformed group means using maximum likelihood estimation; standard errors of logarithm-transformed means were used as weights. The five treatment groups were included as fixed effects in the model. The dependence between multiple means from the same studies was captured through the inclusion of study-specific random effects. Combined effects were estimated and the resulting differences between the four active treatments (supplementation) and control situation were reported (after back-transformation). A significance level of 0.05 was applied. Effect sizes was characterized as either trivial (0–0.2), small (0.2–0.6) or large (>0.6) (Hopkins et al., 2009). Analyses were carried out using the statistical environment R (R Core Team, 2016) with the extension package “metafor” (Viechtbauer, 2010).

## Results

### Beta-alanine

**Figure 3:**
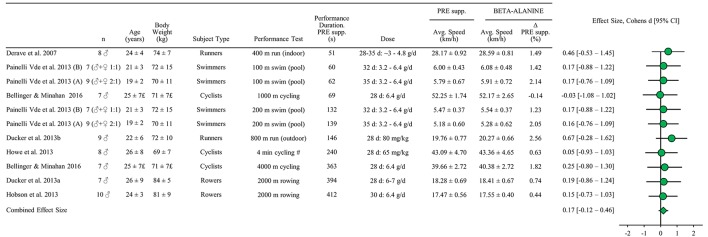
**Standardized mean difference (effect size) with 95% confidence intervals as reported in studies of beta-alanine supplementations effect on performance (average speed) relative to pre supplementation in athletic endurance events lasting 45 s to 8 min**. The combined effect size has been calculated as described in methods section. All other data is reported as mean ± SD. All performance tests were executed indoors on ergometers or treadmills unless stated differently. Regarding Dose: Dose in each study is displayed as days (d) of consumption with the daily dose as gram pr day (g/d). ^#^Speed estimated from mean power reported.^£^Group mean reported (*n* = 14); no info on placebo (*n* = 7) and beta-alanine (*n* = 7) group separately.

A total of 7 studies were identified including 72 subjects. VO_2_-max as an objective measure of training status was reported in two of the studies (Howe et al., 2013; Bellinger and Minahan, 2016). The proportion of female subjects could not be precisely determined due to incomplete reporting of groups in one study (Painelli Vde et al., 2013) but amounted to ~8%. Two studies included several interventions with different performance tests (Painelli Vde et al., 2013; Bellinger and Minahan, 2016). Thus, 11 performance tests were included in the meta-analysis (Figure 3). The estimated combined effect size (with 95% confidence interval) for β-alanine supplementation was 0.17 [−0.12−0.46] which was not significantly different from the pre-supplementation trial (*P* = 0.24).

### Bicarbonate

**Figure 4:**
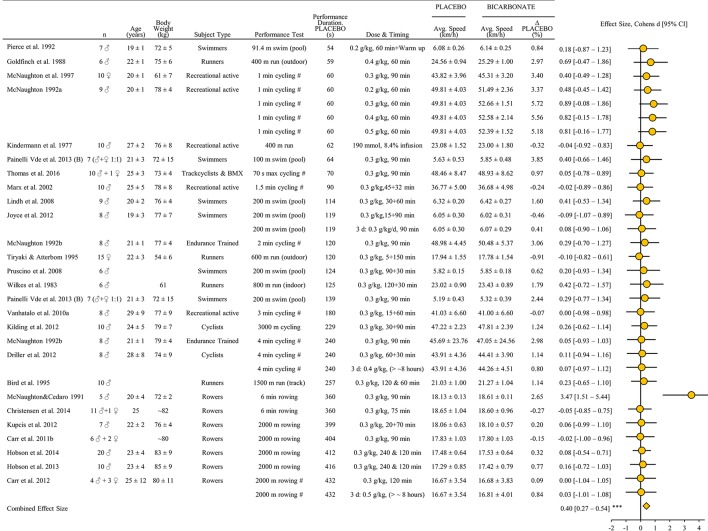
**Standardized mean difference (effect size) with 95% confidence intervals as reported in studies of bicarbonate supplementations impact on performance (average speed) relative to placebo in athletic endurance events lasting 45 s to 8 min**. The combined effect size has been calculated as described in methods section. All other data is reported as mean ± SD. All performance tests were executed indoors on ergometers or treadmills unless stated differently. Regarding Dose & Timing: Dose is listed as gram pr kilo bodyweight (g/kg) and “60 min” denotes time from ingestion to start of test. “30 + 60 min” denotes a 30 min period to consume capsules followed by additional 60 min before start of test and “240 & 120 min” denotes intake of capsules 240 and 120 min before start of test. For studies with chronic loading the amount of days (d) is listed as well as timing of last dose before performance test. ^#^Speed estimated from mean power reported. ^***^Significant effect of bicarbonate on performance (*P* < 0.001).

A total of 25 studies were identified including 235 subjects. VO_2_-max was reported in six of the studies (McNaughton, 1992a,b; McNaughton et al., 1997; Vanhatalo et al., 2010a; Driller et al., 2012; Thomas et al., 2016) and the female subject proportion was ~9%. Six studies included several interventions with different administration of bicarbonate (McNaughton, 1992a; Carr et al., 2012; Driller et al., 2012; Joyce et al., 2012) or evaluation of performance using several tests (McNaughton, 1992b; Painelli Vde et al., 2013). Thus, 33 performance tests were included in the meta-analysis (Figure 4). The estimated combined effect size of 0.40 [0.27–0.54] demonstrates a faster exercise speed after bicarbonate supplementation compared to placebo (*P* < 0.001).

### Caffeine

**Figure 5:**
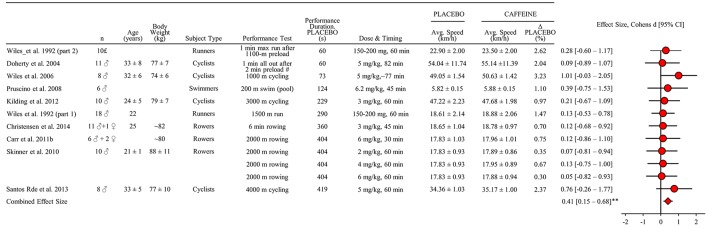
**Standardized mean difference (effect size) with 95% confidence intervals as reported in studies of caffeine supplementations impact on performance (average speed) relative to placebo in athletic endurance events lasting 45 s to 8 min**. The combined effect size has been calculated as described in methods section. All other data is reported as mean ± SD. All performance tests were executed indoors on ergometers or treadmills unless stated differently. Regarding Dose & Timing: Dose is listed as milligram pr. kilo bodyweight (mg/kg) and “60 min” denotes time for consumption before the performance test. ^#^Speed estimated from mean power reported.^£^Four of the 10 subjects were also subjects in part 1 of the study. ^**^Significant effect of caffeine on performance (*P* < 0.01).

A total of 9 studies were identified including 97 subjects. VO_2_-max was reported in three of the studies (Wiles et al., 1992; Skinner et al., 2010; Santos Rde et al., 2013). Assuming only male subjects in protocol “2” by Wiles et al. (1992), the female subject proportion was 3%. One study included several interventions with different administration of caffeine (Skinner et al., 2010) and one evaluated performance using several tests (Wiles et al., 1992). Thus, 12 performance tests were included in the meta-analysis (Figure 5). The estimated combined effect size of 0.41 [0.15–0.68] demonstrates a faster exercise speed after ingesting of caffeine compared to placebo (*P* = 0.002).

### Nitrate

**Figure 6:**
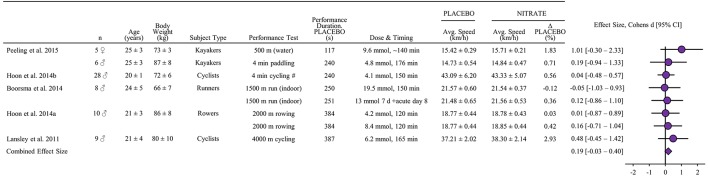
**Standardized mean difference (effect size) with 95% confidence intervals as reported in studies of nitrate supplementation impact on performance (average speed) relative to placebo in athletic endurance events lasting 45 s to 8 min**. The combined effect size has been calculated as described in methods section. All other data is reported as mean ± SD. All performance tests were executed indoors on ergometers unless stated differently. Regarding Dose & Timing: Dose is listed millimoles (mmol) and “140 min” denotes time for consumption before the performance test. ^#^Speed estimated from mean power reported.

A total of 5 studies were identified including 66 subjects. VO_2_-max was reported in three of the studies and 8% of subjects were females. Three studies included several interventions with different administration of nitrate (Boorsma et al., 2014; Hoon et al., 2014a,b) and one study both manipulated administration and exercise testing (Peeling et al., 2015). Thus, 8 performance tests were included in the meta-analysis (Figure 6). The estimated combined effect size of 0.19 [−0.03–0.40] tended to be different from placebo (*P* = 0.09).

### Correlations

**Figure 7:**
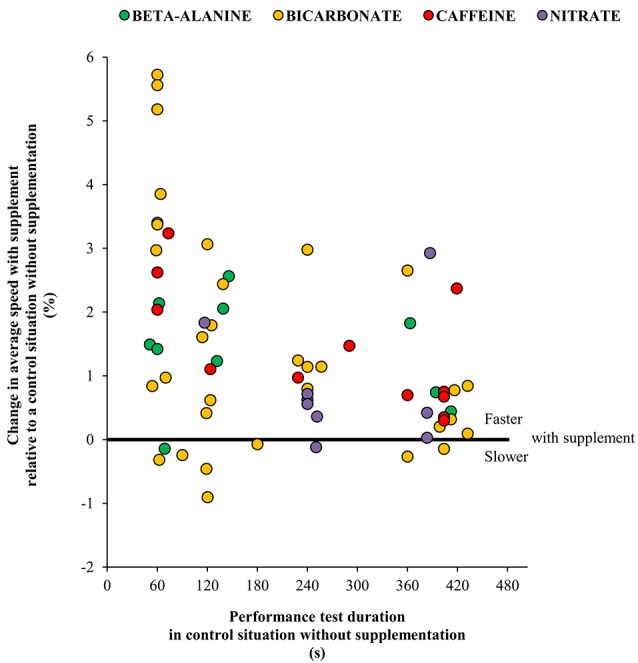
**Association between performance test time and supplement induced change in performance (average speed in athletic events lasting 45 s to 8 min)**. Supplements investigated was beta-alanine (*r*^2^ = 0.07), bicarbonate (*r*^2^ = 0.1922, *P* < 0.05), caffeine (*r*^2^ = 0.45, *P* < 0.05) and nitrate (*r*^2^ = 0.0001).

For all data pooled a modest association was observed between performance test time and percentage improvement (*r*^2^ = 0.17, *P* < 0.001) implying slightly greater performance gains with reductions in performance test time (Figure 7). A similar observation was found for bicarbonate (*r*^2^ = 0.19, *P* < 0.05) and caffeine (*r*^2^ = 0.45, *P* < 0.05), while no association were seen for beta-alanine (*r*^2^ = 0.07) and nitrate (*r*^2^ = 0.0001).

## Discussion

**Figure 8:**
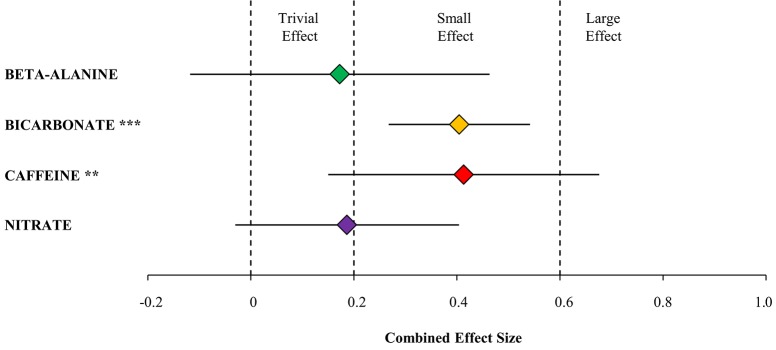
**Combined effect size with 95% confidence interval displaying the impact on performance (average speed) in athletic endurance events lasting 45 s to 8 min from supplementation with either beta-alanine (7 studies) relative to pre supplementation, bicarbonate (25 studies), caffeine (9 studies) or nitrate (5 studies) relative to placebo**. Hatched lines display boundaries for effect sizes being trivial (<0.2), small (0.2–0.6) or large (>0.6). ^***^Significant effect of bicarbonate on performance (*P* < 0.001). ^**^Significant effect of caffeine on performance (*P* < 0.01).

The primary finding in the present meta-analysis is that a small yet significant effect on performance was observed by prior supplementation with caffeine (Effect size, ES = 0.41) or bicarbonate (ES = 0.40) resulting in a higher average speed during closed-end intense endurance exercise tests lasting 45 s to 8 min. No significant performance effect was detectable for supplementation with beta-alanine or nitrate, with both supplements having a trivial effect (ES = 0.17–0.19) (Figure 8).

### Beta-alanine

Based on the 11 performance tests from the 7 included studies with a total of 72 subjects the present analysis demonstrates that beta-alanine supplementation cannot be concluded to be ergogenic in competitive intense endurance sports (Figures 3, 8). This is in contrast to the position stand of the international society of sports nutrition where it is stated that beta-alanine supplementation generally enhances intense exercise lasting more than 60 s (Trexler et al., 2015). In this context, it is important to note that this was primarily found for open-end exercise tests and not the competition relevant time trial test. In line with the conclusion from the present analysis, Hobson et al. (2012) also reported no significant performance effect of beta-alanine supplementation despite improved time to exhaustion based on mixed exercise protocols and Saunders et al. (2017) found that the effect of beta-alanine was ~5 times as high in open-end tests as in performance relevant closed-end tests.

Due to the long supplementation period (~1 month) a concern with all beta-alanine studies is to discriminate between effects from the supplementation *per se* and time effects (e.g., higher or lower training status during the supplementation period). The use of a placebo group or a cross-over design is required to discriminate between effects of supplementation per see or time-effects. Accordingly, in one of the included studies, Derave et al. (2007) reported similar improvement in the placebo and beta-alanine group implying that the changes in performance in the former group could be attributed to other factors than the supplement.

It is unclear how beta-alanine supplementation should improve human exercise performance but the most often suggested possibilities are a higher muscle carnosine content which may increase intramuscular buffer capacity (Parkhouse et al., 1985), increase calcium sensitivity and/or increase calcium release (Dutka et al., 2012) or improve anti-oxidant capacity (Kohen et al., 1988; Pavlov et al., 1993). The paresthethic effect of beta alanine occurring in 0% (Derave et al., 2007; Hobson et al., 2013), ~15% (Bellinger and Minahan, 2016), ~25% (Howe et al., 2013), or ~50 % of participants (Painelli Vde et al., 2013) in the included studies can be speculated to cause a placebo-effect. Still, performance gains around 1% as observed in many of the individual studies (Figures 3, 7) is considered relevant to explore for high performance intense endurance athletes (Table 1) irrespective of the mechanism(s). At present larger studies of elite athletes are required and/or studies of individual responses that can be reproduced which in turn may be affected by factors such as gender, diet and muscle fiber type distribution (Derave et al., 2010). Taken together, the present meta-analysis underlines that no clear ergogenic effect of beta-alanine supplementation can be expected in competitions lasting from 45 s to 8 min.

### Bicarbonate

Bicarbonate supplementation significantly improved performance. The analysis was based on a total of 33 performance tests from 25 studies with a total of 235 subjects (Figures 4, 8). The present finding is in agreement with the recommendation of using bicarbonate as an ergogenic compound when competing in events lasting 1–10 min (Carr et al., 2011a). Contrary to the comprehensive analysis of Carr et al. (2011a) we only included performance relevant tests by excluding open-end test protocols, which is considered of great practical importance for athletes since time to exhaustion testing may introduce larger variability because of motivational and mental aspects (Jeukendrup et al., 1996; Laursen et al., 2007).

It is important to note that exclusion of just one performance test (of a total of 33 tests with 235 subjects)—originating from the study of 5 rowers by McNaughton and Cedaro (1991) having a substantial high effect size (Figure 4)–causes a marked change of both the *p*-value (from below 0.001 to 0.06) and the combined effect size (from 0.40 to 0.19). Accordingly, a balanced interpretation of the data included in the analysis is warranted. Nevertheless, the potential for bicarbonate to improve intense endurance performance seems apparent albeit small, both with (small combined effect size being highly significant different from placebo) or without (combined effect size trivial close to small tending to be different from placebo) inclusion of that particular study.

As with beta-alanine supplementation, bicarbonate's mechanism of action is not fully established but possibly includes increased blood and/or muscle buffer capacity leading to improved protection of muscular contractility via reduced intra- and extracellular H^+^ accumulation (Raymer et al., 2004), potentially affecting calcium sensitivity (Nelson and Fitts, 2014), improved potassium handling (Street et al., 2005), diminished arterial desaturation (Nielsen et al., 2002) or reduced type III/IV afferent firing (Amann et al., 2015). In turn, reductions in blood pH and bicarbonate prior to exercise is known to impair subsequent rowing performance following ammonium chloride ingestion (Brien and McKenzie, 1989) and one legged knee extensor exercise capacity following prior intense arm exercise (Bangsbo et al., 1996; Nordsborg et al., 2003). Thus, the typical increase in blood pH of ~0.1 and bicarbonate concentration of ~5 mM with supplementation (Carr et al., 2011a) may be sufficient to postpone fatigue mechanisms during intense endurance exercise. Based on simple regression analysis, bicarbonate may be more ergogenic in shorter (e.g., 1 min) than longer (e.g., 6 min) performance tests (Figure 7). Nevertheless, the weak association illustrates that more studies are needed, for example including several performance tests of varying duration (e.g., 1, 4, and 8 min) executed by the same subjects. It should be noted that marked individual differences may exist. For example, bicarbonate supplementation caused gastro intestinal problems in 4 out of 21 subjects prior to a time to exhaustion test and exclusion of these four participants resulted in a significant increased exercise time during intense cycling exercise (Saunders et al., 2014). Additionally, there appear to be marked individual differences in bicarbonate uptake kinetics (Jones et al., 2016) and high level athletes may benefit from an individualized protocol (Miller et al., 2016) but this also needs to be addressed in future studies.

### Caffeine

Average speed in intense endurance performance tests lasting 45 s to 8 min is increased by caffeine supplementation. The finding is based on 12 different performance tests from 9 studies with a total of 97 subjects (Figures 5, 8). In half of the studies the improvement in speed exceeded 1% relative to placebo. This observation is in line with a previous narrative review highlighting caffeine as an ergogenic compound for speed endurance events lasting 1–3 min (Davis and Green, 2009) as well as reviews that focus on longer duration exercise lasting several hours (Doherty and Smith, 2004; Ganio et al., 2009). Thus, the present meta-analysis adds to the existing knowledge by establishing that caffeine is also ergogenic in the time domain from 45 s up to 8 min. The present result is in apparent contradiction with the notion that caffeine is less ergogenic in short relative to long-term exercise tests (Doherty and Smith, 2004). However, the previous meta-analysis included both open and closed end exercise testing and different time intervals. Therefore, the discrepancy may be related to the strict selection criteria in the present study, yet both studies outline the ergogenic potential from caffeine in multiple types of exercise tasks. The present meta-analysis does not allow for an evaluation of the interaction between exercise time and caffeine's effect. However, a simple regression analysis based on the non-weighed average effect of the included studies suggests that a larger ergogenic effect may be expected during shorter as compared to longer intense exercise within the time domain of 45 s to 8 min (Figure 7). However, more systematic studies on the possible interaction between exercise duration and magnitude of effect are required before conclusions can be drawn.

Several possibilities exist regarding caffeine's ergogenic effect. These include antagonizing binding of adenosine to its brain receptors which may cause reduced perception of effort and increased arousal and/or peripheral inhibition of muscle pain (Davis and Green, 2009). In support of this notion, participants following caffeine intake report lower perceived exertion during exercise with the same absolute exercise intensity (Doherty et al., 2004; Miller et al., 2014) and in 5 min intervals a similar level of perceived exertion despite a higher mean power with caffeine (Lane et al., 2013). In line herewith, the same degree of exertion has been reported between placebo and caffeine trials during intense endurance performance tests despite improved performance in the latter condition (Santos Rde et al., 2013; Christensen et al., 2014). However, the performance enhancing effect of caffeine may also be related to improved muscle contractility caused by reduced K^+^ accumulation during intense exercise (Mohr et al., 2011) or even by the augmented adrenergic response to caffeine supplementation associated with a larger glycolytic energy turnover during intense endurance exercise (Jackman et al., 1996) although this has been challenged as the main mechanism for the ergogenic effect (Davis and Green, 2009). The exact mechanism of action in specific exercise situations may differ and remains to be elucidated. Caffeine doses in the included studies ranged from 2 to 6 mg/kg body weight, which is in accordance with previous guidelines (Spriet, 2014). Taken together, caffeine appears to improve exercise performance lasting 45 s to 8 min.

### Nitrate

Nitrate supplementation did not significantly improve average speed in the 8 performance tests extracted from the 5 included studies with a total of 66 subjects (Figures 6, 8). Accordingly, previous meta-analyses have reported unclear effects in performance tests with a large range in exercise time (Hoon et al., 2013) or with a mix of protocols (Braakhuis and Hopkins, 2015). However, a tendency (*P* = 0.09) for an effect was identified, underpinning the importance of future well-controlled studies designed to identify small effects (i.e., <1% on average speed) and/or repeatable individual responses. It is noteworthy that nitrate supplementation does not appear to exert any physiologic or endurance performance enhancing effects on group level in highly trained endurance athletes with an average VO_2_-max exceeding 70 ml/min/kg (Peacock et al., 2012; Christensen et al., 2013; Boorsma et al., 2014; Porcelli et al., 2015). Moreover, in a cross sectional study encompassing VO_2_-max values between 28 and 81 ml/min/kg, VO_2_-max explained more than 75% of the magnitude of improvement in performance during a 3 km run (Porcelli et al., 2015). In line herewith, the largest percentage performance effect with nitrate supplementation in the present analysis is observed in the recreational active cyclists undertaking a 4,000-m time trial (Lansley et al., 2011). Interestingly, highly trained kayakers competing at international level experienced improved performance after nitrate supplementation (Peeling et al., 2015) implying that training status probably is not the sole determinant of nitrates ergogenic potential. Important factors may include high or low dietary nitrate intake from vegetable consumption (Jonvik et al., 2016), initial plasma levels of nitrite (Christensen et al., 2017) and muscle fiber composition in the engaged muscles since rat studies demonstrate a larger effect on blood perfusion after nitrate supplementation in muscles with a large proportion of fast twitch fibers (Ferguson et al., 2013).

Nitrate (${\text{NO}}_{3}^{-}$) supplementation increases bioavailability of nitrite (${\text{NO}}_{2}^{-}$) and has been found to lower energy expenditure during exercise and lower resting blood pressure (Larsen et al., 2007), likely due to higher levels of nitric oxide (NO) affecting muscle mitochondria and the circulation (Jones, 2014). The trained human is characterized by a high endogenous capacity for NO production (McConell et al., 2007; Nyberg et al., 2012) which likely is a large contributing factor as to why nitrate supplementation exerts modest physiologic effects in highly trained individuals (Peacock et al., 2012; Christensen et al., 2013; Boorsma et al., 2014; Porcelli et al., 2015). Still reports exist of “responders” in groups of trained (Wilkerson et al., 2012) and highly trained endurance athletes (Christensen et al., 2013; Boorsma et al., 2014) in endurance events besides the report on world class kayakers by Peeling et al. (2015). Thus, future well-designed investigations of possible individual effects of nitrate supplementation are required. At present nitrate supplementation does not appear to have a clear ergogenic effect on intense endurance performance, and the possibility to achieve an ergogenic effect seems greater for moderately trained subjects compared with highly trained endurance athletes.

### Methodologic considerations

It is of importance to consider that a publication bias may exist in the field of ergogenic supplements as it may be easier to publish studies demonstrating performance enhancing effects as compared to studies with no effect that are often under powered to exclude that an effect may exist. We terminated our study search in April 2016 but the field of ergogenic supplements continues to evolve. Recently, studies has been published showing no effect on intense endurance performance from both bicarbonate (Callahan et al., 2016) and nitrate (Callahan et al., 2016) supplementation whereas caffeine has resulted in both unchanged (Cordingley et al., 2016) and improved performance (Boyett et al., 2016). A number of the included studies in the current meta-analysis also reported negative findings, so to what extent an actual publication bias toward positive results exits remains unknown. Nevertheless, reporting of negative results are encouraged in order to provide a balanced and hence accurate foundation for future meta-analyses.

Importantly, the current quality assessment of the included studies displayed a low risk of bias, apart from reporting drop-outs and environmental factors and to a lesser extent time of day for testing and activity the day prior testing (Figure 2) which future studies are encouraged to report.

Other important considerations are the potential impact of age, training status and gender for the observed effects of various supplements on performance. The range in average age among all of the included studies investigated was 19–33 years which provides high ecologic validity for athletes competing at the highest international level (e.g., Olympics). Still, further studies are required to clarify if other effects can be observed in athletes aged for example 30–40 years or even older. Regarding training status, only ~30% of the studies reported maximal oxygen uptake as an objective index of cardio-respiratory capacity and little information was provided about training history. Thus, albeit relevant as highlighted for the nitrate studies, the few studies reporting objective measures of training status preludes an analysis of the general impact of training status. Several studies report their participants to be “trained,” “well-trained,” or “elite” but the used criteria is unclear. An additional challenge in comparing training status, for example based on reported power outputs, is that different methods of obtaining power is known to yield different results. In cycling for instance, differences of 10 W or more at ~400 W is not uncommon (Duc et al., 2007; Kirkland et al., 2008). Nevertheless, an impression of performance level can be obtained by comparing the individual studies performance speed (Figures 3–6) with the velocities of top competitors at the Olympics provided in Table 1. Finally, it is evident that most studies in the present meta-analysis have been conducted with male subjects (Figures 3–6) as the proportion of female subjects did not exceed 10% for any of the four supplements. Thus, it is of relevance to investigate female subjects to a greater extent in future studies. Included studies dominated by females observe both large (McNaughton et al., 1997) and minor (Tiryaki and Atterbom, 1995) effects following bicarbonate intake while large effects also are seen after nitrate supplementation (Peeling et al., 2015).

### Perspectives

In the present meta-analysis, a trivial effect on intense endurance performance was found for beta-alanine (ES = 0.17) and nitrate (ES = 0.19) while small and significant ergogenic effects was found for caffeine (ES = 0.41) and bicarbonate (ES = 0.40). This does highlight that only marginal gains can be expected from supplementation with the four substances (Figure 8). In turn, it is important to note that youth athletes (Eisenmann et al., 2001; Tønnessen et al., 2015) and mature sub-elite athletes (e.g., Esfarjani and Laursen, 2007; Young et al., 2014) in regards to endurance performance can benefit more from physiologic training and maturation rather than supplementation.

Still, supplementation is considered highly relevant for high performance athletes for whom very small margins separate medal rankings during competition (Table 1). Based on the results from the meta-analysis, caffeine and bicarbonate are considered the primary supplements for intense endurance athletes but even a trivial effect from beta-alanine and nitrate may provide an advantage during competition. Accordingly, athletes are encouraged to an individual approach in order to obtain knowledge regarding what supplement(s) has the greatest effect on performance.

Relatively few of the included studies in the present analysis combined use of several supplements with combinations present for caffeine and bicarbonate (Pruscino et al., 2008; Carr et al., 2011b; Kilding et al., 2012; Christensen et al., 2014) and beta-alanine and bicarbonate (Hobson et al., 2013; Painelli Vde et al., 2013). This could be the scope for future studies and help highlight if physiologic interactions between supplements exists and also holds practical importance for high level athletes and staff. Results are mixed for combined administration of caffeine and bicarbonate. Accordingly, using a traditional statistical approach (significance *P* < 0.05) both supplements improved 4000 m cycling performance relatively to placebo but the combination of both did not lead to a greater performance than with single supplementation (Kilding et al., 2012). In rowing caffeine both with and without bicarbonate improved performance (Christensen et al., 2014), unlike observations using magnitude-based inferences statistics where bicarbonate abolished the improvement in performance with caffeine alone (Carr et al., 2011b). Adding bicarbonate to beta-alanine supplementation has been reported to improve 2,000 m rowing (Hobson et al., 2013) as well as 100 and 200 m swimming performance (Painelli Vde et al., 2013) with magnitude-based inference statistics, but not with traditional statistics (Painelli Vde et al., 2013). In a recent meta-analysis including a mix of exercise protocols beta-alanine together with bicarbonate induced a significant effect relative to use of beta-alanine alone (Saunders et al., 2017). Clearly more studies are needed to establish if positive or negative interactions between supplements are present.

Legal supplements other than the four analyzed in the present meta-analysis exist that may also impact on intense endurance performance. Creatine has been investigated in a few studies with reports of both unchanged (Burke et al., 1996; Mujika et al., 1996; Syrotuik et al., 2001; De Andrade Nemezio et al., 2015) and improved performance (Rossiter et al., 1996; McNaughton et al., 1998). Importantly the majority of these studies reported mean power from ergometers (Rossiter et al., 1996; McNaughton et al., 1998; Syrotuik et al., 2001; De Andrade Nemezio et al., 2015) but for athletes in which body mass often is a concern it is important to account for any increase in weight which is common with creatine supplementation (Mujika et al., 1996; McNaughton et al., 1998; De Andrade Nemezio et al., 2015). Accordingly, an increased body weight will increase resistive forces on an athlete during forward propulsion leading to a higher energy expenditure for a given speed especially during running (LeCheminant et al., 2009) and in the water (Mujika et al., 1996).

Another supplement is the antioxidant n-acetyl cysteine (NAC) which following oral intake did not alter a 2–5 min time-trial performance in one study (Slattery et al., 2014) but enhanced exercise capacity in time to exhaustion tests lasting ~8 min in another study (Corn and Barstow, 2011) but more studies are needed to establish if oral intake of NAC may be performance enhancing.

In light of the central actions that caffeine exerts during exercise lowering perception of effort (Doherty et al., 2004; Lane et al., 2013; Santos Rde et al., 2013; Christensen et al., 2014; Miller et al., 2014) the use of analgesic substances not banned at present may also exert an ergogenic effect. This is partly supported by improved time-trial performance (lasting ~26 min) and similar perception of effort as with placebo in trained cyclists after intake of 1,500 mg acetaminophen (Mauger et al., 2010) which is a mild over-the-counter analgesic in most countries. In cycling, the presence of the morphine like drug Tramadol in ~5% of all samples analyzed in 2015 by the World Anti-Doping Agency (WADA) also seems to indicate that performance enhancement may be present with administration of analgesic substances (WADA, 2015). This area is poorly explored and confer ethical considerations even though the net response of a reduced perception of effort appears similar to caffeine, albeit by different methods and with different potential side effects (Doherty et al., 2004; Lane et al., 2013; Miller et al., 2014).

For future studies we encourage use of individual data and ideally repeated use of a given supplement to better discriminate between day-to-day variation and true performance effects for the individual subject/athlete. This may also shed light on potential mechanisms for a supplement (not) to work if combined with other measures such as dietary habits, gender, muscle fiber distribution, mitochondrial capacity, buffer capacity, lactate production and adrenergic response during exercise.

## Conclusion

Using a meta-analytic approach with a strict focus on “closed-end” (e.g., time-trials or fixed duration) intense endurance performance tests lasting from 45 s to 8 min, prior intake of caffeine or bicarbonate demonstrated a small positive effect on average speed being significantly different from placebo. Nitrate had a trivial effect on performance and weakly tended to be faster than placebo, but at present nitrate appears most relevant for non-elite athletes or athletes with modest aerobic power. Lastly beta-alanine effects on intense endurance performance was trivial. For all supplements, individualized approaches are advised since factors such as diet, gender, supplement uptake kinetics, muscle oxidative capacity and fiber distribution may impact on whether an ergogenic effect can be obtained.

## Author contributions

PC and NN: Design of study, data analysis, interpretation of results, manuscript draft. YS: Data analysis, revision of manuscript. CR: Design of study, data analysis, revision of manuscript.

### Conflict of interest statement

The authors declare that the research was conducted in the absence of any commercial or financial relationships that could be construed as a potential conflict of interest.
